# Facing COVID-19 in times of armed conflicts in Northern and Central regions of Mozambique

**DOI:** 10.1057/s41271-021-00300-2

**Published:** 2021-09-08

**Authors:** Agostinho Viana Lima

**Affiliations:** Department of Letters and Social Science, University of Rovuma, Niassa, Mozambique

**Dear Editors,**

Mozambique is facing two great adversaries. One is COVID-19 that claimed 132 confirmed deaths and 15,866 infections in its first round since the first case recorded on 22 March 2020 until 2 December 2020 [1]. The second is the armed conflicts in the Northern region of Mozambique perpetrated by armed forces linked to the *Jihadist* group known as *Al-Shabaab*, and in the Central region by self-proclaimed Military Junta linked to RENAMO (Mozambique Nacional Resistance, the major opposition party). From October 2017 to October 2020, these insurgents conducted over 600 terrorist attacks in the Northern and Central districts of Cabo Delgado province, causing about 2000 deaths, over 60% among civilians [2]. By the end of September 2020, the violence increased internal displacement to over 300,000 people, equivalent to 13% of the population [2].

Despite the ongoing conflicts and displacement, the rate of infection of COVID-19 in Mozambique initially remained low compared to neighboring countries. On 2 December 2020, South Africa had recorded 792,299 cases of COVID-19 and 21,644 deaths [3]. In Zambia, there were 17,665 cases and 357 deaths. In Zimbabwe and Malawi, 277 and 185 deaths were reported [3], respectively. According to the World Health Organization (WHO), the low counts of COVID-19 in Mozambique could be due strict preventive measures [4].

Eight days after the first recorded case, president of Mozambique, Filipe Jacinto Nyusi, approved a Presidential Decree (No 11/2020) declaring a state of emergency [5]. During the first 30 days, preventive measures included: limiting contacts, stay-at-home advisory, prohibiting public or private events, and suspending all school activities, from nurseries to universities. The decree required execution of State of Emergency measures be ensured by the Defense and Security Forces. The government designated both law and police to enforce compliance. Non-compliance would be considered a crime of disobedience with penalties of imprisonment of up to 6 months and a steep fine, an equally strong penalty [5, 6]. After 21 days of the first State of Emergency, at least 400 people had been detained and one killed; several more had been injured for non-compliance [7]. President Filipe Nyusi criticized police violence in imposing the state of emergency [8].

Later, the President extended the state of emergency three times. Enforcement of later States of Emergency switched from military to municipal and local civilian authorities. Defense and the Security Forces were to act only in special circumstances. Non-compliance continued to be a crime of disobedience punishable with a penalty of 3–15 days in prison, or by a fine, or community service. Yet, more cases of disobedience were reported. Furthermore, epicenters of COVID-19 transmission in Maputo City and Maputo province emerged. Between 16 June and 2 December 2020, in Maputo City and province cases rose from 83 to 8,275 and from 60 to 2,655 cases, respectively.

The third State of Emergency still required the mandatory home quarantine of 14–21 days for all citizens returning to the country, mandatory use of masks, travel restrictions, and restrictions for public and private events [9]. On 3 June 2020, the Mozambican local newspaper *Noticias* reported that about 30 people had been detained daily in Maputo province for violating the state of emergency and ignoring measures to prevent the spread of infections [10]. According to the National Health Institute, from the start of the pandemic on 22 March 2020 to 07 July 2021 Maputo City had reported the most positive cases of COVID-19 (34,361 cases), as compared to central provinces, such as Sofala (5,328 cases), Manica (2,826 cases), and in the north, such as Cabo Delgado (3,486 cases) and Nampula (3,283 cases) [3].

Some non-compliance also occurred in the north, attributable mainly to the armed conflict in Cabo Delgado that caused massive population displacement. In just two years, from the end of 2018 until 19 October 2020, in Cabo Delgado and the Central region the numbers of displaced due to intensified armed conflict rose from 15,000 to 424,202—an increase of over 2,700% [2]. It is estimated that 670,000 people have been forced to leave their homes owing to the armed conflict in Cabo Delgado [11]. The Portuguese newspaper *RTP Notícias* stated that the official number of displaced is around 700,000 people [12] and the number of displaced is increasing in Pemba (the Cabo Delgado provincial capital) as armed attacks continue to intensify. With more insurgent attacks in several districts, including Mocímboa da Praia, Macomia, and Quissanga e Muidumbe, the migration increased from about 100,000 displaced in February 2020 to 172,186 in April, and 211,485 in May of 2020, reaching about 10% of the province’s population [13, 14]. The government was completely unprepared to handle both migration and pandemic crisis [2].

As migration increased in Cabo Delgado, in May 2020 the health authorities declared the city of Pemba and Nampula province to be epicenters of community transmission. Even then, some reception centers sheltered numerous families in a single tent, without masks [13]. People were exposed to both coronavirus infection and malaria, unable to follow preventive measures, struggling to find food and the minimum necessities to survive [2]. Many were forced to work informally [14]. In Mozambique, the informal sector contributes about 60% to the Gross Domestic Product (GDP) and employs around 80% of working population [15]. The informal workers have no provision of free supply of masks and for many masks are unaffordable [14].

Inadequate availability of health care is a barrier to achieving health as a right [16]. The ratio of medical and nurse workers to population remains very low (55 per 100,000 habitants) compared to 230 per 100,000 habitants recommended by WHO [17]. The Government of Mozambique recognizes many challenges including lack of access to health services, shortage of health professionals, and weak infrastructure [18]. The epidemiologic situation of the northern provinces of Cabo Delgado and Nampula and the central provinces of Sofala and Manica reflect the limited availability of health care and the sub-notification of COVID-19 cases [19].

The combined crisis of the COVID-19 pandemic, armed conflicts, migration, and displacement threatens to overwhelm the government of Mozambique. Lacking the resources and infrastructure to confront these complex challenges, the country continues to see an increase in the numbers of displaced families from the northern and central regions and the increase in positive cases of COVID-19. The Government’s additional challenges include increased shortage of health professionals and unemployment. In response to increased cost of living, the country is experiencing an increase of families involved in informal trade and, consequently, non-compliance to the prevention measures of COVID-19 decreed by the Government. The weak infrastructures means that this public health crisis could worsen. For this reason, it is important that the Mozambiquan Government define and enact steps to ensure peace and social and economic inclusion.
